# The Role of Clinical, Plasma, and Imaging Biomarkers in Assessing Future Dementia Risk in Individuals With Subjective Cognitive Decline

**DOI:** 10.1212/WNL.0000000000214983

**Published:** 2026-05-14

**Authors:** María Rivera Sánchez, Sophie E. Mastenbroek, Shorena Janelidze, Pontus Tideman, Niklas Mattsson-Carlgren, Danielle Van Westen, Erik Stomrud, Oskar Hansson, Sebastian Palmqvist, Rik Ossenkoppele

**Affiliations:** 1 Clinical Memory Research Unit, Department of Clinical Sciences Malmö, Faculty of Medicine, Lund University, Sweden;; 2 Department of Neurology, Marqués de Valdecilla University Hospital, Santander, Cantabria, Spain;; 3 University of Cantabria, Santander, Spain;; 4 IDIVAL Health Research Institute, Santander, Cantabria, Spain;; 5 Amsterdam Neuroscience, Brain Imaging, the Netherlands;; 6 Department of Radiology and Nuclear Medicine, Vrije Universiteit Amsterdam, Amsterdam University Medical Center, location VUmc, the Netherlands;; 7 Memory Clinic, Skåne University Hospital, Malmö, Sweden;; 8 Wallenberg Center for Molecular Medicine, Lund University, Sweden;; 9 Diagnostic Radiology, Department of Clinical Sciences, Lund University, Sweden;; 10 Image and Function, Skåne University Hospital, Lund, Sweden;; 11 Amsterdam Neuroscience, Neurodegeneration, the Netherlands; and; 12 Alzheimer Center Amsterdam, Neurology, Vrije Universiteit Amsterdam, Amsterdam UMC location VUmc, the Netherlands.

## Abstract

**Background and Objectives:**

Subjective cognitive decline (SCD) is a well-recognized risk state for developing mild cognitive impairment (MCI) and dementia. Optimal risk stratification for early interventions and clinical trial selection remains challenging. This study evaluates progression risk across multimodal biomarker profiles in SCD.

**Methods:**

We conducted a longitudinal observational study including participants from the BioFINDER-1 and BioFINDER-2 cohorts with a baseline diagnosis of SCD, at least 1 follow-up visit, and available information on dementia progression. Baseline predictors included cognitive performance, *APOE4* status, plasma phosphorylated tau (p-tau) 217, “AD-signature” cortical thickness, hippocampal volume, and white matter hyperintensities (WMHs) measured by the Fazekas scale. Missing data were handled using multiple imputation. Predictors were evaluated individually and then combined in progressively complex Cox regression models to predict progression to all-cause dementia, Alzheimer disease (AD) dementia, and MCI (BioFINDER-2 only). Model performance was assessed using the Harrell C-index, and Akaike information criterion was used for comparing model fit.

**Results:**

A total of 469 participants with SCD (mean age 69.1 ± 7.1 years, 51.4% female) were included in the main sample. Eighty-four individuals progressed to dementia over 4.0 ± 2.1 years (66.7% AD dementia). Progressors were older and more frequently *APOE4* carriers and showed worse baseline cognition, higher plasma p-tau217, and greater atrophy and WMH burden. Plasma p-tau217 was the strongest individual predictor for AD dementia (C-index = 0.86 ± 0.012), but multivariable models outperformed single-biomarker models. The best model for all-cause dementia included all variables and achieved a C-index of 0.89 ± 0.003. For AD dementia, a more parsimonious model combining plasma p-tau217, cognitive scores, and APOE4 status showed excellent predictive ability (C-index = 0.91 ± 0.009), with only marginal improvement when MRI markers were added. Among 249 individuals from BioFINDER-2, 84 progressed to MCI within 2.3 ± 1.2 years. For MCI prediction, model performance was generally lower and similar between the plasma model and the model including all variables (C-index = 0.83 ± 0.009).

**Discussion:**

A clinically feasible multimodal approach combining cognitive assessment, plasma p-tau217, and *APOE4* status accurately predicts AD dementia risk in individuals with SCD. Adding MRI measures of brain atrophy and WMHs further improves prediction for all-cause dementia. These findings underscore the clinical value of plasma p-tau217 in refining risk assessment in SCD and support its potential implementation in memory clinic settings alongside other widely available biomarkers.

## Introduction

Subjective cognitive decline (SCD) refers to self-reported decline in cognitive functioning in any given domain, without objective evidence of impairment on standard neuropsychological test batteries. In 2014, the Subjective Cognitive Decline Initiative (SCD-I) identified SCD as a preclinical stage with a higher risk of future Alzheimer disease (AD) dementia,^1^ and longitudinal studies have supported increased risk of progression to mild cognitive impairment (MCI) and dementia^2-6^ in SCD. This recognition has gained even more relevance after the recent shift in AD diagnostic criteria toward a biological definition of the disease using imaging or biofluid biomarkers of β-amyloid (Aβ) plaques and tau neurofibrillary tangles.^7^ Identifying SCD profiles most susceptible to cognitive decline is essential for clinical guidance, selecting candidates for early interventions, and enrolling at-risk individuals in trials of disease-modifying therapies.

Previous research has identified multiple risk factors that can help predict clinical progression in individuals with SCD. Subtle cognitive impairments, particularly in memory and executive function, can be detected through standard neuropsychological tests, such as the Alzheimer's Disease Assessment Scale–Cognitive Subscale, Trail-Making Tests A and B, and animal fluency. These measures have been shown to predict future cognitive deterioration when assessing preclinical and early stages, including cognitively unimpaired individuals at risk of AD and individuals with SCD.^4,8-14^ Genetic factors also contribute to cognitive decline risk, with the *APOE4* allele being the most well-established genetic contributor to AD susceptibility. Its presence has been linked to an increased risk of cognitive decline,^15,16^ particularly when accompanied by subjective memory reports.^17^ In the past years, growing evidence has supported the role of CSF biomarkers of AD pathology, mainly Aβ1–42 and phosphorylated tau (p-tau), in early diagnosis and prognosis of cognitive decline.^18,19^ A recent meta-analysis^3^ showed that these CSF biomarkers can predict the risk of future cognitive decline and progression to MCI or dementia in individuals with SCD. More recently, blood biomarkers have emerged as accessible and reliable tools for early AD detection, particularly plasma p-tau217,^20-22^ and have been incorporated into the current AD diagnostic guidelines proposed by the Alzheimer's Association Workgroup.^7^ While studies in SCD populations are limited, emerging evidence suggests that blood biomarkers may help identify individuals at higher risk of cognitive decline and progression to dementia in this group.^4,12^ In addition, structural MRI also provides important prognostic information. Reduced cortical thickness in AD-vulnerable regions, smaller hippocampal and amygdalar volumes, and vascular changes reflected by a higher burden of white matter hyperintensities (WMHs) have been linked to an increased risk of progression to MCI/dementia in preclinical and early stages of neurodegenerative dementias, including SCD.^12,23-29^

Despite growing evidence supporting the prognostic value of these biomarkers, there is a scarcity of large-scale studies involving SCD cohorts with extended follow-up periods, particularly those incorporating blood biomarkers alongside clinical and biological markers to predict dementia risk. The aim of this study was, therefore, to determine whether clinical and biological markers, including demographic factors, baseline cognitive performance, *APOE* genotype, and plasma and MRI biomarkers, are associated with dementia risk in individuals with SCD from the BioFINDER-1 and BioFINDER-2 cohorts. Through comprehensive multivariable analyses, we evaluated different combinations of these predictors to identify the most accurate and robust models for assessing future risk of all-cause dementia, AD dementia, and MCI in individuals with SCD.

## Methods

### Participants

Participants were recruited from the BioFINDER-1 (NCT01208675) and BioFINDER-2 (NCT03174938) cohorts. These are memory clinic-based studies conducted at Skåne University Hospital and Ängelholm Hospital in southern Sweden, where individuals are referred as part of routine clinical practice. Both BioFINDER-1^4^ and BioFINDER-2^30^ are prospective longitudinal cohorts, with annual follow-up visits, including repeated clinical, cognitive, neurologic, and psychiatric assessment. Further details have been published previously.^31^ For this study, we selected individuals diagnosed with SCD during the baseline visit. Inclusion and exclusion criteria for participants with SCD in the BioFINDER studies can be found in eMethods.

A total of 547 individuals with SCD were recruited by December 2024 (222 from BioFINDER-1 and 326 from BioFINDER-2). Inclusion in the main analysis required available information on dementia progression and at least 1 follow-up visit. After applying these criteria, the final sample consisted of 469 individuals with SCD (eFigure 1). Participants were also evaluated for progression to MCI. This outcome was evaluated only in BioFINDER-2 because this information was not available in BioFINDER-1. A total of 249 BioFINDER-2 participants met the inclusion criteria and constituted the subsample for assessing MCI progression risk.

### Predictors

#### Cognition

We selected cognitive tests representing episodic memory and executive/attention domains that were available in both the BioFINDER-1 and BioFINDER-2 cohorts and have been shown to be more robustly linked to clinical progression in individuals with SCD and cognitively unimpaired individuals.^8-14^ Details on composites construction are provided in eMethods.

#### *APOE* Genotype

Genotyping for *APOE* (gene map locus 19q13.2) was performed using previously described methods.^32^ Genotypes were determined based on 2 single-nucleotide variants, rs7412 and rs429358, which define the ε2, ε3, and ε4 alleles, and dichotomized as carrier of at least 1 ε4 allele (*APOE4*) vs noncarrier.

#### Plasma p-tau217

Plasma p-tau217 was measured from blood samples collected at baseline. Concentrations were quantified at the Memory Research Unit using an immunoassay developed by Lilly Research Laboratories and run on the MSD platform, according to methodology described in previous studies.^33^

#### Brain Atrophy and WMH Burden

At baseline visit, all participants underwent MRI on a 3T scanner. The BioFINDER MRI protocol has been described previously.^34^ Cortical thickness across 68 brain regions of interest (ROIs) from the Desikan-Killiany atlas was measured using FreeSurfer version 6.0.^35^ Cortical thickness was also extracted from the predefined “AD-signature” region, comprising temporal regions vulnerable to early AD atrophy, including the bilateral entorhinal cortex, inferior temporal gyrus, middle temporal gyrus, and fusiform gyrus (adjusted for surface area).^36^ Volumetric measures of the amygdala and hippocampus were obtained using Sequence Adaptive Multimodal SEGmentation (SAMSEG) in FreeSurfer version 7.1 and normalized to total intracranial volume. To account for co-occurring vascular pathology, WMHs were further assessed. We selected the Fazekas visual scale, given its widespread use and established applicability in clinical practice. An experienced neuroradiologist evaluated WMHs on axial FLAIR images using the Fazekas scale, which separately grades periventricular and deep WMHs (0–3; higher scores indicating greater burden). Following previous studies, scores were dichotomized into absent/mild WMHs (Fazekas 0–1) and moderate-to-severe WMHs (Fazekas 2–3).^37^ Fazekas scores were used for all primary analyses, while WMH burden quantified using an automated segmentation method (SAMSEG), with volumes normalized to intracranial volume, was examined in a sensitivity analysis.

### Model Development and Statistical Analysis

R software (version 4.5.0) was used for statistical analyses.

We first compared baseline characteristics between participants who progressed to dementia and those who remained stable, using the Mann-Whitney *U* test for continuous variables and the χ^2^ test for categorical variables. Cognitive tests, plasma p-tau217, and MRI measures were *z*-transformed based on a reference cohort (eMethods).

To assess regional brain differences associated with progression to dementia, we performed logistic regression analysis on cortical thickness across the 68 ROIs, as well as on subcortical volumes including the hippocampus and amygdala (details in eMethods).

**Figure 1:**
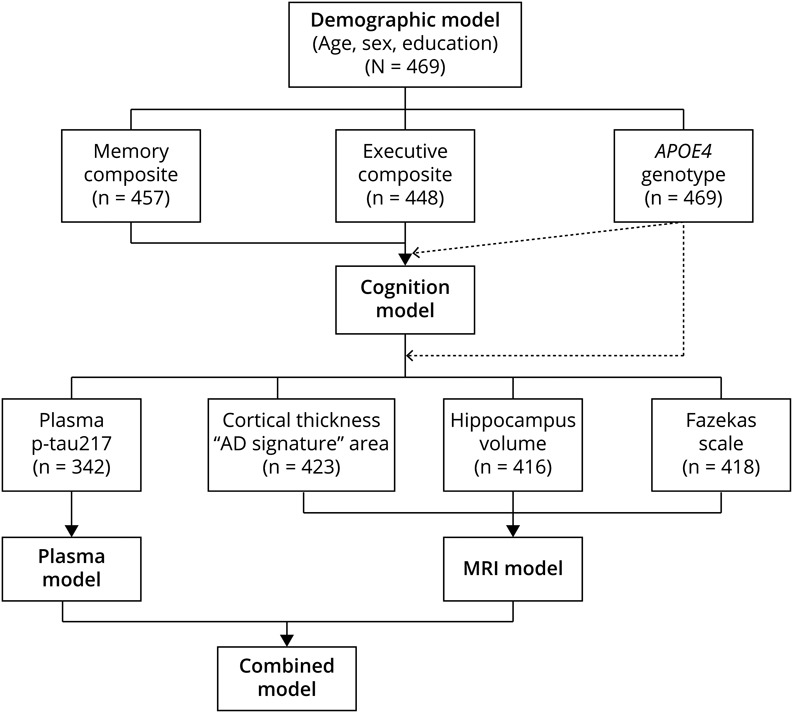
Multivariable Model Development Flowchart Overview of the stepwise development of the multivariable models used to assess the risk of progression to dementia in individuals with subjective cognitive decline. Models increased in complexity through the sequential addition of demographic, cognitive, plasma, and MRI biomarkers. Each n reflects the number of individuals with available data for the specific variable. Each model was also fitted with *APOE4* carrier status as an additional covariate.

The main analysis was conducted using Cox proportional hazards regression models to evaluate associations between demographic variables (age, sex, and years of education), cognitive composites, plasma p-tau217, cortical thickness in the “AD-signature” region, hippocampal volume, and Fazekas score with 3 different outcomes. Main outcome definitions are provided in eMethods. The primary outcomes were progression to all-cause dementia and progression to AD dementia. In a secondary analysis, we applied the same models with progression to MCI as the outcome measure. Numerical predictors were standardized (*z*-scored) during model fitting. To ensure interpretability, *z*-scores for cognitive composites and MRI continuous variables were inverted, so that higher values reflected worse performance or greater atrophy, aligning their directionality with that of plasma p-tau217. The availability of predictors of interest among individuals with SCD from both BioFINDER cohorts is shown in Figure 1. We compared individuals with at least 1 missing value in any variable with those with complete data (eTable 1). Missing values in predictors were handled using multiple imputation by chained equations (further details in eMethods). In the initial analysis, each predictor was evaluated individually for each outcome while retaining age, sex, and years of education as covariates, and hazard ratios (HRs) with 95% CIs were reported. In addition to the prespecified outcomes, an exploratory analysis assessed these predictors for progression to non-AD dementia, to evaluate their specificity for predicting AD vs other dementia subtypes.

Next, we developed a series of progressively complex multivariable models using a stepwise approach, sequentially adding the previously mentioned predictors (Figure 1 and eMethods provide an overview of the different models). Models' performance was assessed using the C-index, summarized as mean ± SD across imputations. The Akaike information criterion (AIC) was also calculated to compare model fit. As a sensitivity analysis, these multivariable models were also tested in complete-case samples, including only individuals with complete data for the variables included in each respective model. Progression to non-AD dementia was not evaluated in the multivariable model approach because of the limited number of events.

Finally, we implemented a stratified K-fold internal cross-validation combined with multiple imputation for the multivariable models. Details of the cross-validation methodology are provided in eMethods.

### Standard Protocol Approvals, Registrations, and Patient Consents

The BioFINDER studies were approved by the Regional Ethics Committee in Lund, Sweden, and all participants provided written informed consent to participate. This study followed the Strengthening the Reporting of Observational Studies in Epidemiology reporting guidelines.

### Data Availability

All relevant source data from this study, along with anonymized data from the BioFINDER studies, will be shared on request by a qualified academic investigator for the sole purpose of replicating procedures and results, provided data transfer complies with European Union Data Protection Regulation and decisions by the Ethical Review Board of Sweden and Region Skåne, regulated through a material transfer agreement. The code for statistical analyses is available at a public repository.

## Results

### Baseline Characteristics and Group Differences

The final sample consisted of 469 individuals with SCD, of whom 84 (17.9%) progressed to dementia. The mean time to dementia diagnosis was 4.0 ± 2.1. AD was the most common diagnosis (n = 56; 66.7%), followed by Lewy body dementia and vascular dementia, each accounting for 9 cases (10.7%). At baseline, 66 individuals (78.6%) who progressed to dementia had an abnormal CSF Aβ42/40 ratio.

**Figure 2:**
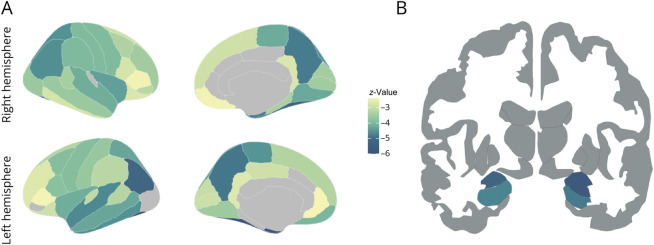
Brain Atlas Representing Differences in Cortical Thickness (A) and Hippocampal and Amygdalar Volumes (B) Between Participants With SCD Who Progressed to Dementia and Those Who Did Not Cortical ROIs are displayed according to the Desikan-Killiany cortical atlas in panel A, while hippocampal and amygdalar volumes are shown on a coronal map in panel B. The color bar reflects the *z*-values obtained from logistic regression models adjusted for age and corrected for multiple comparisons using the FDR method. Regions are considered significant if the FDR-corrected *p* value was <0.05. In panel A, light gray indicates nonsignificant regions; in panel B, dark gray indicates regions not included in the analysis. FDR = false discovery rate; ROI = region of interest; SCD = subjective cognitive decline.

Table 1 summarizes the baseline characteristics of participants with SCD, stratified by progression to all-cause dementia during follow-up. Participants who progressed to dementia were significantly older (72.7 ± 4.8 vs 68.3 ± 8.2 years, *p* < 0.001), had fewer years of education (11.8 ± 3.7 vs 13.1 ± 3.7, *p* = 0.002), and were more often *APOE4* carriers (60.7% vs 40.3%, *p* = 0.001, ε2/ε4 and ε2/ε4 heterozygotes 48.8% vs 34.3%; ε4/ε4 homozygotes 11.9% vs 6.0%). At baseline, progressors demonstrated worse cognitive performance, as reflected by significantly lower Mini-Mental State Examination scores (27.9 ± 1.5 vs 28.7 ± 1.4, *p* < 0.001), memory *z*-scores (−1.27 ± 1.1 vs −0.4 ± 0.9, *p* < 0.001), and executive function *z*-scores (−0.7 ± 0.8 vs −0.2 ± 0.6, *p* < 0.001). In addition, progressors had higher plasma *p*-tau217 levels (*z* = 2.9 ± 3.0 vs 0.6 ± 1.5, *p* < 0.001) and showed lower cortical thickness in “AD-signature” region (*z* = −1.4 ± 1.6 vs −0.2 ± 1.0, *p* < 0.001), smaller hippocampal volume (*z* = −1.0 ± 1.2 vs −0.1 ± 1.0, *p* < 0.001), and a higher prevalence of moderate-to-high WMH burden (Fazekas score 2–3: 53.0% vs 35.1%, *p* < 0.005). An exploratory analysis revealed significantly lower baseline cortical thickness across multiple brain regions and reduced amygdalar and hippocampal volumes in progressors vs nonprogressors to dementia (Figure 2). The most prominently affected cortical areas included the fusiform, inferior parietal, middle temporal, superior temporal, precuneus, paracentral, and entorhinal cortices.

**Table 1:** Baseline Characteristics of Individuals With SCD From BioFINDER-1 and BioFINDER-2 Cohorts

	Converting to dementia (n = 84)	Not converting to dementia (n = 385)	*p* Value
Demographics			
Sex, female, n (%)	38 (45.24)	203 (52.73)	0.261
Age, y	72.66 (4.81)	68.32 (8.18)	<0.001
Education, y	11.81 (3.65)	13.11 (3.66)	0.003
*APOE4* carrier, n (%)	51 (60.71)	155 (40.26)	<0.001
Follow-up data			
Time to any dementia, y	4.04 (2.05)		
Follow-up time in stable participants, y		4.64 (2.61)	
Main diagnosis, n (%)			
Alzheimer disease dementia	56 (66.67)^a^		
Lewy body dementia	9 (10.71)^b^		
Vascular dementia	9 (10.71)		
Frontotemporal dementia	4 (4.76)^c^		
Other dementias	6 (7.14)^d^		
Cognitive scores			
MMSE score	27.95 (1.52)	28.75 (1.38)	<0.001
Memory function, *z*-score	−1.27 (1.05)	−0.36 (0.87)	<0.001
Attention/executive functions, *z*-score	−0.71 (0.77)	−0.22 (0.65)	<0.001
Abnormal CSF Aβ42/40 ratio, n (%)	66 (78.57)	143 (37.14)	<0.001
Plasma p-tau217, *z*-score	2.91 (3.04)	0.57 (1.55)	<0.001
MRI measures			
“AD-signature” region cortical thickness, *z*-score	−1.40 (1.57)	−0.20 (1.05)	<0.001
Hippocampal volume/icv, *z*-score	−1.01 (1.22)	−0.10 (1.02)	<0.001
Fazekas 2–3, n (%)	43/81 (53.01)	119/337 (35.11)	0.005
WMH volume/icv (*z*-score)	0.40 (1.19)	0.27 (1.23)	0.335

Abbreviations: AD = Alzheimer disease; icv = intracranial volume; MMSE = Mini-Mental State Examination; p-tau = phosphorylated tau; SCD = subjective cognitive decline.

Variables are presented as mean (SD) for continuous data or as n (%) for categorical variables. Group comparisons were performed using the Mann-Whitney *U* test for continuous variables or the χ^2^ test for categorical variables.

Memory function and attention/executive functions are composites derived from cognitive tests. Memory function includes the Alzheimer's Disease Assessment Scale immediate and delayed word recall test. The attention/executive composite includes animal fluency, Trail-Making Test A, and Trail-Making Test B.

Cognitive domain scores, plasma p-tau217 levels, and MRI measures were standardized into *z*-scores using linear regression models adjusted for relevant covariates (age for all measures, and education further for cognitive tests), based on a reference population of cognitively unimpaired individuals from BioFINDER-1 and BioFINDER-2 cohorts.

The “AD signature” region includes the entorhinal, inferior temporal, middle temporal, and fusiform regions.

Fazekas scores were dichotomized as 0–1 vs 2–3, representing absent/mild vs moderate-to-severe white matter lesions.

White matter hyperintensity volumes were obtained using automated segmentation with SAMSEG, adjusted for intracranial volume, and then transformed into *z*-scores.

a Among participants who progressed to AD dementia, 4 presented with the logopenic variant of AD and 1 with the posterior cortical atrophy variant.

b Of the individuals with SCD who progressed to suspected Lewy body dementia, 3 had comorbid AD pathology and 1 was considered to have possible limbic-predominant age-related TDP-43 encephalopathy.

c Four participants were diagnosed with frontotemporal dementia spectrum disorders: 2 with the behavioral variant frontotemporal dementia, 1 with the semantic variant primary progressive aphasia, and 1 with the nonfluent variant primary progressive aphasia.

d Other dementias include 1 case of Parkinson disease dementia, 1 case of progressive supranuclear palsy, and 4 cases of nonspecified neurodegenerative dementia.

In the BioFINDER-2 cohort (n = 249), 84 individuals (33.7%) progressed to MCI during follow-up (2.3 ± 1.2 years), with 25 subsequently converting to dementia (3.5 ± 1.4 years). AD was the most common diagnosis (64.3%), followed by prodromal DLB (10.7%) and vascular disease (5.9%). Among progressors to MCI, 79.8% were amyloid-positive in CSF, compared with 38.2% of nonprogressors (*p* < 0.001). The proportion of *APOE4* carriers was not significantly different between groups (53.6% vs 46.7%, *p* = 0.33, ε2/ε4 and ε2/ε4 heterozygotes 46.4% vs 40.0%; ε4/ε4 homozygotes 7.1% vs 6.7%). Progressors to MCI had significantly lower memory scores (*z* = −0.7 ± 0.7 vs −0.2 ± 0.8, *p* < 0.001), while differences in the attention/executive composite did not reach statistical significance (*z* = −0.3 ± 0.6 vs −0.2 ± 0.7, *p* = 0.545). They also exhibited higher baseline plasma p-tau217 levels (*z* = 2.5 ± 2.6 vs 0.3 ± 1.3, *p* < 0.001), greater atrophy (“AD-signature” area cortical thickness: *z* = −0.6 ± 1.1 vs −0.3 ± 0.9, *p* = 0.006; hippocampal volume: *z* = −0.8 ± 1.2 vs −0.2 ± 1.0, *p* < 0.001), and increased WMH burden (moderate-to-high Fazekas score: 49.3% vs 22.0%, *p* < 0.001). eTable 2 summarizes baseline differences between progressors and nonprogressors to MCI.

### Single-Predictor Models

**Figure 3:**
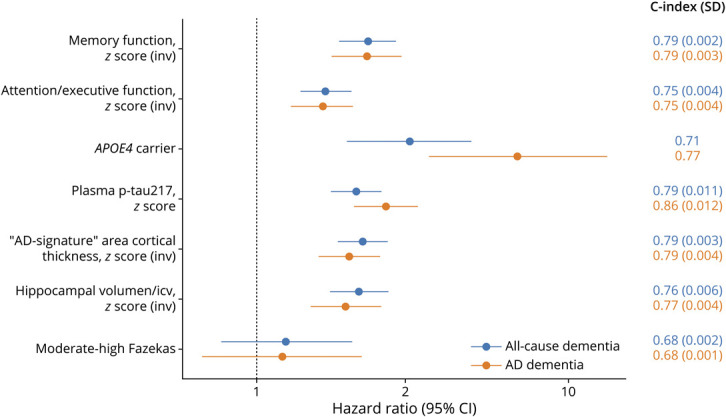
Regression Coefficients and Harrell's C-Indices of Individual Biomarker Models for Assessing the Risk of Progression to All-Cause Dementia and AD Dementia HRs with 95% CIs are shown for each predictor from separate Cox models including 1 biomarker at a time, with age, sex, and education as covariates. Cognitive domain scores, plasma p-tau217 levels, and MRI measures were standardized into *z*-scores using linear regression models adjusted for relevant covariates (age for all measures, and education further for cognitive tests), based on a reference population of cognitively unimpaired individuals from BioFINDER-1 and BioFINDER-2 cohorts. To ensure interpretability, *z*-scores for cognitive and MRI variables were inverted, so that higher values consistently indicated worse performance or greater atrophy, aligning with the direction of plasma p-tau217. All models were fitted in the same sample of individuals with SCD from BioFINDER-1 and BioFINDER-2 cohorts (n = 469), after multiple imputation of missing data. Continuous variables were scaled before imputation. Model performance is indicated by the Harrell's concordance index and SD (C-index [SD]), with the SD reflecting variability across imputations. For *APOE4* genotype, no SD is reported because this variable was complete and did not require imputation. AD = Alzheimer disease; HR = hazard ratio; icv = intracranial volume; (inv) = inverted; p-tau = phosphorylated tau.

We evaluated the predictive performance of individual biomarkers for the development of all-cause dementia and AD dementia, adjusting for demographics, using the multiply imputed data sets. Figure 3 shows the comparison of the HRs and C-index of these models for progression to all-cause dementia and progression to AD dementia. Further information can be found in eTable 3. The same predictors were subsequently assessed for their ability to predict progression to MCI in the BioFINDER-2 sample after multiple imputation of missing data (eTable 4).

#### Single-Predictor Models for All-Cause Dementia

Among all biomarkers, memory composite, plasma p-tau217, and “AD-signature” cortical thickness demonstrated the highest discriminative performance for all-cause dementia risk (C-index = 0.79). Among cognitive measures, memory composite outperformed executive composite, with a HR of 2.28 [95% CI 1.84–2.81] and C-index of 0.79 ± 0.002 vs HR of 1.66 [95% CI 1.38–2.01] and C-index of 0.75 ± 0.004. Hippocampal volume showed a slightly lower discriminative ability than “AD-signature” cortical thickness (C-index = 0.76 ± 0.006 vs 0.79 ± 0.003, respectively). By contrast, moderate-to-high WMH burden exhibited the lowest predictive accuracy (C-index = 0.68 ± 0.002) and was not a significant predictor in the model. Consistent results were observed in a sensitivity analysis using WMH volumes derived by the automated SAMSEG method (eTable 3). Finally, *APOE4* carrier status showed moderate predictive ability for all-cause dementia (C-index = 0.71). Age was consistently significant across all models, whereas sex and education were not significantly associated with dementia risk in most models.

#### Single-Predictor Models for AD Dementia

Plasma p-tau217 had a higher discriminative performance for predicting AD dementia (C-index = 0.86 ± 0.012) compared with prediction in all-cause dementia. *APOE4* carrier status was the predictor with the highest HR (6.86 [3.56–13.22]), and its model yielded a C-index of 0.77. Cognitive composites demonstrated a similar performance as observed for all-cause dementia (memory composite: C-index = 0.79 ± 0.003; executive composite: C-index = 0.75 ± 0.004). Structural MRI measures also showed a good performance, with “AD-signature” cortical thickness reaching a C-index of 0.79 ± 0.004, and hippocampal volume 0.77 ± 0.004. By contrast, the moderate-to-high burden of WMHs measured by the Fazekas scale again exhibited the lowest predictive value (C-index = 0.68 ± 0.001) and was not significantly associated with the outcome, with comparable results obtained from automated segmentation measures (eTable 3).

In comparison, exploratory analysis of these predictors for non-AD dementia risk (eTable 5) showed that *APOE4* carrier status and plasma p-tau217 were not significant predictors of non-AD dementia. Instead, structural MRI measures and cognitive composites were significantly associated with this outcome.

#### Single-Predictor Models for MCI

Similarly, when assessing the risk of progression to MCI, plasma p-tau217 demonstrated the highest discriminative performance among biomarkers (C-index = 0.78 ± 0.011), followed by the memory composite (C-index = 0.75 ± 0.007) and the structural MRI measurements (“AD-signature” region cortical thickness: C-index = 0.71 ± 0.002; hippocampal volume: C-index = 0.71 ± 0.003). Higher WMH burden, represented by Fazekas scores of 2–3, exhibited the lowest performance and was not significantly associated with MCI risk (C-index = 0.68 ± 0.003). Sensitivity analysis using WMH volumes from SAMSEG yielded similar results. *APOE4* carrier status was also not significantly associated with progression to MCI (*p* = 0.13). Age remained a consistently significant predictor across all single-predictor models.

### Multivariable Cox Regression Models

**Figure 4:**
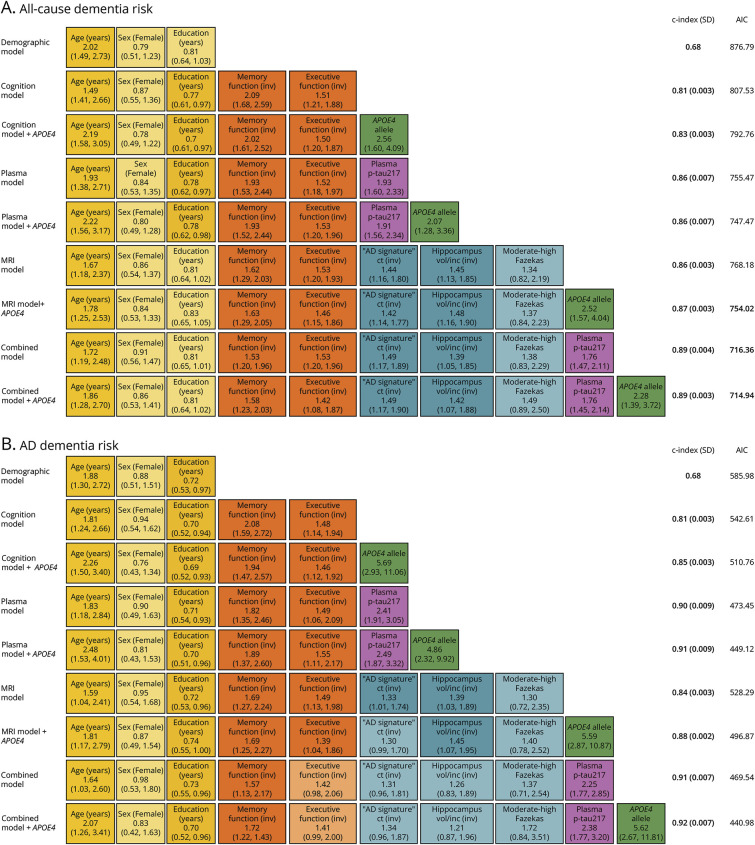
Comparative Performance of Multivariable Models for Assessing the Risk of All-Cause Dementia (A) and AD Dementia (B) in Individuals With SCD Models included different combinations of variables: demographics (age, sex, education), cognitive measures (memory and attention/executive functions), plasma biomarker (p-tau217), *APOE4* allele status, and MRI-based imaging markers (cortical thickness in “AD-signature” region, hippocampal volume adjusted by icv, and Fazekas score for white matter hyperintensities). Cognitive domain scores, plasma p-tau217 levels, and MRI measures were standardized into *z*-scores using linear regression models adjusted for relevant covariates (age for all measures, and education additionally for cognitive tests), based on a reference population of cognitively unimpaired individuals from BioFINDER-1 and BioFINDER-2 cohorts. To ensure interpretability, *z*-scores for cognitive and MRI variables were inverted, so that higher values consistently indicated worse performance or greater atrophy, aligning with the direction of plasma p-tau217. All models were fitted in the same sample of individuals with SCD from BioFINDER-1 and BioFINDER-2 cohorts (n = 469), after multiple imputation of missing data. Continuous variables were scaled before imputation. For each variable in each model, HR with 95% CI is presented. HRs with nonsignificant *p* values are shown using a lighter color in the figure. Model performance is indicated by the Harrell concordance index and SD (C-index [SD]), with the SD reflecting variability across imputations. SD is not reported for the demographic model because all variables included in this model were complete and needed no imputation. AIC is reported as a measure of model fit. Lower AIC values and higher C-index indicate better model performance. AIC = Akaike information criterion; AD = Alzheimer disease; ct “AD-signature” = cortical thickness of the “AD signature” area (this area includes the measurements for entorhinal, inferior-temporal, middle temporal, and fusiform regions); HR = hazard ratio; icv = intracranial volume; (inv) = inverted; p-tau = phosphorylated tau; vol = volume; SCD = subjective cognitive decline.

Model performance was first assessed after multiple imputation of missing data for all-cause dementia and AD dementia risk. Figure 4 illustrates the performance of the different multivariable models for these outcomes. Results from sensitivity analysis, in which models were tested on complete-case data, are provided in eTable 6. The same approach was applied to evaluate model performance for progression to MCI in BioFINDER-2 (eFigure 2 and eTable 7).

#### Multivariable Cox Regression Models for the Risk of All-Cause Dementia

The demographic model showed modest discriminative ability for all-cause dementia (C-index = 0.68), with age as the only variable significantly associated with dementia risk. The cognition model demonstrated good performance (C-index = 0.81 ± 0.003), with memory function emerging as the strongest predictor (HR 2.09, 95% CI 1.68–2.59, *p* < 0.001), while executive function showed a lower HR (HR 1.51, 95% CI 1.21–1.88, *p* < 0.001). The plasma model exhibited robust predictive performance (C-index = 0.86 ± 0.007), with plasma p-tau217 identified as a strong predictor (HR 1.93, 95% CI 1.60–2.33, *p* < 0.001). The MRI model similarly showed high discriminative ability for assessing all-cause dementia risk (C-index = 0.86 ± 0.003); both structural MRI measures were significantly associated with risk, whereas moderate-to-high Fazekas scores did not show a significant association. The combined model achieved the highest predictive accuracy (C-index = 0.89 ± 0.003). Plasma p-tau217 (HR 1.76, 95% CI 1.47–2.11, *p* < 0.001) and memory function (HR 1.53, 95% CI 1.20–1.96, *p* < 0.001), together with age, were the main contributors to model performance. Overall, model performance and model fit slightly improved when adding *APOE4* carrier status as a covariate, with this predictor showing a significant association with dementia risk across the different models. The best-fitting model, based on the lowest AIC, was the combined model with *APOE4* as the covariate (AIC = 714.94); however, excluding *APOE4* resulted in only a minimal increase in AIC (716.36).

#### Multivariable Cox Regression Models for the Risk of AD Dementia

For assessing the risk of AD dementia, the demographic and the cognition models showed a similar performance to that observed for the prediction of all-cause dementia (C-index = 0.68 and 0.81, respectively). The plasma model demonstrated excellent predictive performance (C-index = 0.90 ± 0.009), with a slight improvement with the addition of *APOE4* carrier status as a covariate, reaching a C-index of 0.91 ± 0.009. The MRI model also showed good performance (C-index = 0.84 ± 0.003). In this model, structural MRI measures were significantly associated with risk, while moderate-to-high Fazekas scores were not a significant covariate. When all predictors were included in the combined model, the C-index increased to 0.92 ± 0.007. In this model, *APOE4* carrier status (HR 5.62, 95% CI 2.67–11.81, *p* < 0.001) and plasma p-tau217 (HR 2.38, 95% CI 1.77–3.20, *p* < 0.001) were the strongest predictors, whereas the structural MRI measurements lost statistical significance. The lowest AIC was again observed for the combined model with *APOE4* carrier status as the covariate (AIC = 440.98); however, the plasma model plus *APOE4* showed a comparable model fit (AIC = 449.12) and a similar discriminative ability (C-index = 0.91 ± 0.009 vs 0.92 ± 0.007).

#### Multivariable Cox Regression Models for the Risk of MCI

Overall, model performance for MCI prediction was generally lower than for dementia outcomes, although the combined model achieved good discriminative ability (C-index = 0.83 ± 0.009). The predictors most strongly and consistently associated with MCI progression were plasma p-tau217, memory composite, and age. Among MRI markers, hippocampal volume remained significantly associated across models, while cortical thickness in the “AD-signature” region and moderate-to-high Fazekas scores were not significantly associated with MCI risk. In general, *APOE4* status did not significantly contribute to model performance. When comparing the models for MCI risk, the combined model and the plasma model demonstrated similar discriminative ability (C-index = 0.83), although the combined model showed a slightly better fit (AIC = 720.4 vs 724.06) (eFigure 2 shows further details).

#### Internal Cross-Validation

**Figure 5:**
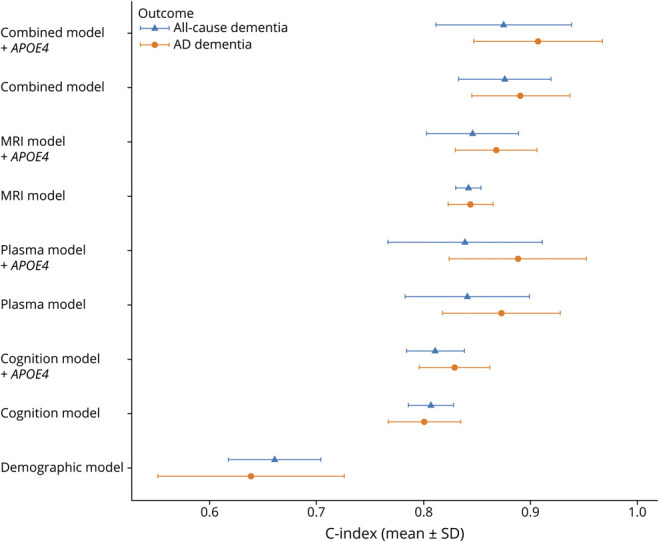
Internal Cross-Validation Performance of Multivariable Models for Assessing the Risk of All-Cause Dementia and AD Dementia in Individuals With Subjective Cognitive Decline Points represent the mean C-index across 5 stratified folds, with error bars showing ±1 SD. Cox regression models were trained using multiple imputation for missing values in the training set, and missing values in the test set were imputed using simple mean/mode imputation. Higher C-index values reflect better discrimination of individuals who progress to dementia. AD = Alzheimer disease.

Internal 5-fold cross-validation of the multivariable models was performed to assess the robustness of our results. Overall, models showed better performance in predicting progression to AD dementia (Figure 5). Variability in C-index between folds was higher for models including plasma p-tau217, likely because of the greater number of missing values handled through multiple imputation. For AD dementia risk, models incorporating plasma p-tau217 outperformed MRI models, reaching a C-index of 0.89 (0.82–0.95) when *APOE4* carrier status was added. This was slightly lower than the model combining all variables, which reached a C-index of 0.91 (0.85–0.97).

In the case of MCI risk (eFigure 3), overall models showed lower performance than for dementia outcomes, potentially reflecting the smaller sample size. The model comparison was consistent with the main analysis, except that the C-index values were lower. The 2 best performing models were the plasma model (C-index = 0.79 [0.76–0.83]) and the combined model (C-index = 0.78 [0.76–0.80]), and the addition of *APOE4* carrier status did not improve model performance.

## Discussion

In this study, we conducted survival analyses to evaluate the risk of clinical progression in individuals with SCD from the BioFINDER-1 and BioFINDER-2 cohorts. We studied different predictors, including memory and executive/attention tests, plasma p-tau217, *APOE4*, MRI atrophy measurements (cortical thickness of the “AD-signature” area and hippocampal volume), and WMH burden, evaluating their individual and combined associations with future risk of progression to 3 different outcomes: all-cause dementia, AD dementia, and MCI. Our findings indicate that a clinical model incorporating memory and executive tests, plasma p-tau217, and *APOE4* genotype can accurately predict the risk of progression to AD dementia. The addition of established structural MRI measurements did not significantly enhance the model's predictive value for AD dementia but did improve prediction for all-cause dementia. When applied in a subgroup of individuals from BioFINDER-2, this multimodal biomarker combination demonstrated moderate performance in assessing MCI risk.

Our results support that a relatively simple and clinically feasible multimodal approach, based on widely used memory and executive function tests, plasma p-tau217, and *APOE4* genotype, can accurately predict the risk of future dementia, particularly AD dementia, in individuals with SCD. Our findings are consistent with previous research and reinforce the central role of plasma p-tau217 as a highly informative early biomarker of AD-related pathologic changes.^4,38^ It is important to note that this study also supports the specificity of plasma p-tau217, which was not significantly associated with progression to non-AD dementia in the exploratory analysis. When assessed individually, plasma p-tau217 achieved a C-index of 0.86 ± 0.012 for predicting future AD dementia. Its combination with memory and executive function composites and *APOE4* genotype yielded excellent performance, with a C-index reaching 0.91 ± 0.009. These findings align with a previous subanalysis in a smaller subsample of individuals with SCD, where a model combining plasma p-tau217, *APOE4*, and similar cognitive composites predicted AD dementia over 4 years with an area under the curve of 0.95.^4^ These results also support *APOE4* as a predictor of progression to dementia, particularly AD dementia, aligning with its well-established association with future cognitive decline.^6,15,17^ However, in our study, *APOE4* was not associated with an increased risk of MCI, contrasting with findings from some previous studies in cognitively unimpaired *APOE4* carriers.^39,40^ This discrepancy may be due to the smaller sample size in the MCI analysis subgroup, which could have limited statistical power, as well as the heterogeneity previously reported in MCI.^41^ This may also explain the lower predictive performance of the models for MCI risk, evidenced by decreased performance in the internal cross-validation. Our findings further align with literature linking early brain atrophy, particularly cortical thinning in the AD-signature region and reduced hippocampal volume, to increased dementia risk.^4,25^ Notably, in our cohort, the predictive value of MRI-derived structural measures diminished in models that incorporated plasma p-tau217, especially for AD prediction, supporting the hypothesis that plasma p-tau217 may reflect very early pathophysiologic changes,^42^ possibly preceding detectable structural neuroimaging changes, as previously proposed.^12^ For all-cause dementia, however, the combined model, including MRI measurements, improved model fit. Furthermore, when exploring “AD signature” cortical thickness and hippocampal volume as predictors of non-AD dementia, both measures remained significantly associated with dementia risk, suggesting that structural imaging retains value in predicting broader dementia risk beyond AD, capturing non–AD-related pathologies, as suggested by previous literature.^43^ The other MRI marker, WMH burden as measured by the Fazekas score, did not show a significant association with dementia or MCI outcomes across the different models. Similarly, WMH volumes derived from automated segmentation using SAMSEG were not significantly related to dementia or MCI risk in Cox regression analyses adjusted for demographic variables. In the main sample, 32% of individuals with SCD who progressed to AD dementia had MRI-based evidence of vascular copathology, with baseline differences reaching statistical significance for Fazekas scores. We hypothesize that the effect of WMH on cognitive decline may be attenuated in the multivariable models by other predictors, such as age, which itself showed a significant association with dementia and MCI risk.

Identifying biomarkers capable of detecting individuals with SCD at risk of cognitive progression is essential. Growing evidence of increased risk of cognitive decline in SCD has led recent guidelines to classify individuals with SCD in the context of confirmed amyloid pathology within stage 2 of the AD continuum,^7^ distinguishing them from stage 1 individuals who report no cognitive concerns. Recognizing this distinction underscores the need to analyze participants with SCD separately in research studies and clinical trials, especially in memory clinic cohorts, where higher progression rates in SCD have been reported compared with community-based cohorts.^6,44^ A recent study from the BioFINDER-2 cohort,^45^ including individuals with SCD and healthy controls, demonstrated that individuals with SCD had an approximately fivefold higher rate of progression to MCI than healthy controls and exhibited higher baseline plasma biomarker levels. Notably, the percentage of phosphorylated to nonphosphorylated tau217 (%p-tau217) was a strong predictor of progression to MCI in both groups. These findings further support the use of biomarker testing in selected cases with suspicion of AD, in line with the recommendations of Alzheimer's Association CSF biomarker guidelines.^46^

A key strength of this large-scale longitudinal study is its focus on a well-defined memory-clinic cohort of individuals with SCD, allowing for a detailed investigation of plasma p-tau217 and its combination with clinical and imaging biomarkers in predicting the risk of progression to dementia or MCI. Unlike previous research with mixed cohorts of SCD, MCI, or cognitively unimpaired older adults, this study uniquely examines these associations within a large SCD-only cohort. However, some limitations should be acknowledged. The primary limitation is the absence of an external validation cohort to assess the generalizability of our findings. In addition, the MCI risk analysis was based only on the BioFINDER-2 cohort, resulting in a smaller sample and lower statistical power, so the findings should be interpreted with caution. Furthermore, cognitive assessment was restricted to memory and executive domains, potentially underrepresenting cognitive functions relevant to other dementia types, such as language and visuospatial abilities. Finally, unavailable data on ethnicity and socioeconomic status may restrict generalizability and prevent evaluation of their potential impact on progression. Future research should aim to include SCD cohorts with longer follow-up, enable individual-level risk prediction, and use larger samples for MCI progression analyses. Furthermore, exploring additional blood-based biomarkers, such as neurofilament light chain, the Aβ42/40 ratio, or glial fibrillary acidic protein, in combination with plasma p-tau217, may enhance predictive accuracy and provide a more comprehensive understanding of risk profiles in SCD.

## Glossary

Aβ: β-amyloid
AD: Alzheimer disease
AIC: Akaike information criterion
HR: hazard ratio
MCI: mild cognitive impairment
p-tau: phosphorylated tau
ROI: region of interest
SAMSEG: Sequence Adaptive Multimodal SEGmentation
SCD: subjective cognitive decline
SCD-I: Subjective Cognitive Decline Initiative
WMH: white matter hyperintensity
